# Differential Cellular Effects of Electroporation and Electrochemotherapy in Monolayers of Human Microvascular Endothelial Cells

**DOI:** 10.1371/journal.pone.0052713

**Published:** 2012-12-27

**Authors:** Cécil J. W. Meulenberg, Vesna Todorovic, Maja Cemazar

**Affiliations:** 1 Faculty of Health Sciences, University of Primorska, Izola, Slovenia; 2 Department of Experimental Oncology, Institute of Oncology Ljubljana, Ljubljana, Slovenia; Tel-Aviv University, Israel

## Abstract

*In vivo* electroporation of tumours shows disruption of blood flow and creates a vascular effect with an initial rapid and transient vasoconstriction phase and a much longer lasting phase with changed microvascular endothelium. These changes are not well understood but are presumed to involve the cytoskeleton. The paper presents for the first time differential *in vitro* effects describing cytoskeleton changes and monolayer integrity changes by both electroporation and electrochemotherapy of monolayers of human microvascular endothelial cells (HMEC-1). After the application of electric field pulses, the morphology of cells, and both the F-actin and Beta-tubulin cytoskeleton proteins were affected. During both electroporation and electrochemotherapy, the initial phase of cellular damage was noticed at 10 min as swollen cells and honeycomb-like actin bundles. The electroporation-induced cellular effects, observed from electric pulses >150 V, were voltage-dependent and within 24 hrs partly recoverable. The electrochemotherapy-induced cellular effects developed at 2 hrs in spindle-like cells, and more densely packed F-actin and Beta-tubulin were observed, which were dependent on the amount of bleomycin and the voltages applied (>50 V). In addition, for electrochemotherapy with electric pulses >150 V cellular changes were not recoverable within 24 hrs. The effects on monolayer integrity were reflected in the enhanced monolayer permeability, with the electrochemotherapy showing an earlier onset and synergy. We conclude that electrochemotherapy as compared to electroporation leads within 24 hrs to a quicker and more pronounced monolayer integrity damage and endothelial cell death, which together provide further insight into the cellular changes of the vascular disruption of electrochemotherapy.

## Introduction

The technique of electroporation (EP) facilitates cellular gene and drug delivery for agents that initially have no or limited transmembrane transport [1]–[3]. Besides, *in vivo* electroporation shows disruption of blood flow and creates a vascular effect consisting out of an initial rapid and transient vasoconstriction phase, followed by a much longer lasting phase resulting in changed microvascular endothelium [4]–[6]. All together the blood flow is modified locally and without systemic effects [5], [6]. This occurs simultaneously with an increase of the blood vessel permeability and improves the delivery of intravenous injected molecules to specific tissue- and/or intracellular targets [4], [6], [7]. Combined treatment of EP with chemotherapeutic drugs, i.e. electrochemotherapy (ECT) is now in routine clinical practice for treatment of subcutaneous tumours of different histology, predominantly melanomas [1], [2], [8]. ECT is used mainly as palliative treatment of painful and bleeding metastases, where its vascular effect is beneficial, as the bleeding stops immediately after the treatment [9], [10].

The use of several natural toxins like cytochalasin and latrunculin that inhibit actin filament polymerization, and colchicine that inhibits microtubule polymerization, has shown the importance of the actin/tubulin cytoskeleton network in (1) the electroporation effectiveness [11]–[14] and (2) the plasmid expression during gene electrotransfer therapies [15], [16]. Besides, changes in the endothelial barrier function by the re-modeling of the endothelial cytoskeleton were suggested to contribute to the vascular disrupting actions of electroporation [17]. Immediately after EP, cultured human umbilical vein endothelial cells (HUVECs) showed a profound voltage-dependent disruption of actin filament and microtubule cytoskeletal networks, loss of cadherin cell junctions and a rapid increase in monolayer permeability [17]. A similar disruption of the tubulin networks was shown for fibroblasts and Chinese hamster ovary cells, however in these cells no alteration of the actin cytoskeleton was observed [11], [18]. These effects were reversed within 1 hour after EP. In contrast, high electric field (*E*) drastically reduced the cell viability and cytoskeletal renewal within the next 24 hours [17].

The knowledge of possible cytoskeletal and morphologic changes of endothelial cells due to ECT is of importance in controlling the *in vivo* ECT-induced vascular effect and needs to be better understood in order to take advantage of the ECT-induced vascular effect more effectively. The aim of our study was thus first to establish an *in vitro* endothelial cell system in which cultured adherent endothelial cells of microvascular origin were used to mimic tumour endothelium, and secondly to evaluate the ECT-induced changes in endothelial cell monolayer permeability, cell morphology and cytoskeletal proteins to further elucidate mechanisms involved in ECT-induced vascular disruption in tumours.

## Materials and Methods

### Cell culture

HMEC-1s were cultured in MCDB-131 supplemented with 10% foetal calf serum, 1% glutamax (all from Gibco, USA), and 500 µg/L hydrocortisone, 5 µg/L epidermal growth factor, 15 mg/L gentamicin (Sigma Aldrich, USA) and subcultured on 12-well inserts with diameter of 1.4 cm having 3 µm diameter pores at a density of 2×10^6^/cm^2^ (ThinCerts, Greiner Bio One, Germany) or in 8 chamber slides of each 0.75 cm×0.95 cm (Lab-Tek, Nunc, Denmark). The monolayers were allowed to reach confluence and used for experiments starting 2 to 3 days after subculture.

### Electroporation and electrochemotherapy

Culture medium was removed and the monolayers were washed with phosphate buffer saline (PBS, Gibco, USA) and subsequently equilibrated in EP buffer containing 125 mM sucrose, 10 mM K_2_HPO_4_, 2,5 mM KH_2_PO_4_, 2 mM MgCl_2_·6H_2_O. Electrode positioning of parallel plate electrodes (gap = 7.3 mm, width = 7.3 mm) was perpendicular to the monolayer and enabled to electroporate 47% and 75% of the monolayer for the inserts and chambers respectively. A single 1 Hz train of 8 pulses each 100 µs in length was applied to each monolayer with *E* of either: 0, 50, 100, 200, 300, 400, 500 V/7.3 mm electrode gap (corresponding with 0, 68, 137, 274, 411, 548, 685 V/cm respectively) without bleomycin (EP) or in the presence of 3, 30, 300 or 1000 nM bleomycin (ECT). Each slide contained a control chamber with a non-electroporated monolayer. After the therapy, the cells were stationary in the EP buffer for 5 minutes, in order to equilibrate and make both EP and ECT effective. This was followed by a PBS wash, after which, depending on the experiments, the PBS was either replaced with culture medium, or the slide was prepared for staining. In the chamber slides the efficiency of single HMEC-1 cell membrane permeation during EP was monitored by evaluation of the cellular uptake of the fluorescent propidium iodide (PI, Sigma Aldrich).

### Endothelial monolayer permeability assay

Once the endothelial monolayers were formed on the ThinCerts, the monolayers were extensively washed with PBS and checked for exhibiting tight barrier properties by addition of medium containing 0.5 mg/mL fluorescein isothiocyanate (FITC)-coupled dextran (70 kDa molecular weight, Sigma Aldrich). Samples of medium from inserts and wells were collected after 30 minutes and fluorescence was measured at 485/515 nm by a fluorescence microplate reader (Tecan, Austria). In the case of monolayers exhibiting tight barrier properties, there was no leakage of FITC-dextran to the wells, consequently no fluorescence was detected in the wells. EP/ECT was performed as described above with the addition that immediately after treatment the inserts were placed in wells containing fresh medium, and the EP buffer in the inserts was replaced by medium containing FITC-coupled dextran. At specific time points (either at 30 s, 1 min, 5 min, 10 min, 20 min, and 30 min) three samples of 100 µL were collected and the fluorescence was measured. The permeability of the monolayer correlates with the fluorescence intensity in the wells, which for each treatment was normalized to the fluorescence intensity of control monolayers. The normalized fluorescence intensity was fitted by a three-parameter sigmoidal curve using SigmaPlot (Systat Software Inc., USA), and the slope of the initial linear part of the curve, correlating with the rate of permeability, was determined by linear regression using the least square method.

### Giemsa and immunofluorescence staining

Chamber slides with monolayers were prepared for either Giemsa or immunofluorescence at 10 min, 2 hrs, and 24 hrs post-EP/ECT. For Giemsa staining, the MCDB-131 culture medium was removed, and the monolayers were washed with PBS, air dried, fixed in cold methanol, stained with fresh Giemsa solution (Merck, Germany), and rinsed with tap water. Slides for immunofluorescence were washed with PBS, fixed in 3.7% formaldehyde (Sigma Aldrich), permeabilised in 0.1% triton (Sigma Aldrich) and washed with PBS. Next, the monolayers were incubated for 30 min with FITC-conjugated phalloidin (1∶40, F432, Invitrogen, USA) or 90 min incubation with monoclonal mouse anti-B tubulin (1∶500, 18-0093, Invitrogen, USA), followed by a 60 min incubation with a FITC-conjugated anti-mouse IgG (1∶30, F2012, Sigma Aldrich, USA). Between all the immunofluorescence steps the monolayers were extensively but carefully washed with PBS. After staining, the slides were stored at 4°C. Next day, mounting solution containing PI (Vectashield, Vector Laboratories, USA) was applied to stain cytosolic and nuclear RNA/DNA.

### Microscopy and image analysis

Slides were analysed and pictures were taken with a NIKON Eclipse 80i microscope equipped with B2A and G2A filters, a digital camera (DXM1200F) and ACT-1 software (all from Nikon, USA). Details of cell morphology, cytoskeleton and nuclei were evaluated and characterized using both high and low magnification objectives (4× and 40×, Plan Fluor, Nikon) in combination with 10× ocular eyepieces. The observations of one slide were taken under identical microscopic lightning conditions. Images were saved at a resolution of 3840×3072 pixels and were inverted and converted to grayscale using the ImageJ software (NIH, USA). Subsequently the threshold tool was used: (1) to determine the fractional black and white pixel distribution and yielded data for the cellular material covered surface area, (2) to analyse the distribution of the sizes of the particles present in the image. The thresholds for determining the covered surface area were generally lower than the threshold for the determination of particles, where border particles were excluded. Each slide contained one chamber with a monolayer not electroporated. From these control conditions, the number of viable HMEC-1 cells in the picture, were determined to correspond to threshold particles amounting to surface areas (in pixel^2^) of circular shapes. From control slides in which all the 8 chambers were initially plated from the same cell suspension, both the values for the covered surface area and the number of cells, were reproducible with high accuracy and with little deviation within the chambers of a single slide (data not shown, maximum standard error 7%).

### Statistics

The data points are presented as means ± standard error, with *n* the number of independent experiments, and are calculated as percentages from the control non-electroporated monolayer parameters. One-way repeated analysis of variance (ANOVA) was used to make inter-group comparisons. If significance was reached, a Bonferroni post hoc t-test was used to compare individual groups. A value of *P*<0.05 was used as being significant. All presentations of results, curve fitting and statistical analyses were performed with SigmaPlot (Systat Software Inc., USA).

## Results

### Efficiency of electroporation

**Figure 1:**
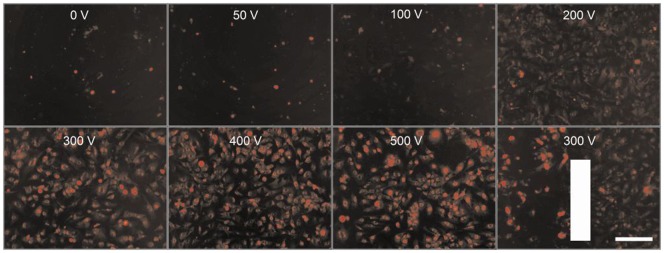
Efficiency of electroporation in HMEC-1 monolayers. Pictures of monolayers were taken within 3 min post-EP in electroporation buffer containing 100 µM PI. The vertical bar indicates the position of the right parallel electrode: area to the left within the *E* of 300 V and area to the right outside the electric field. Bar = 200 µm.

We assessed whether the present experimental setup was suited to generate efficient EP conditions, i.e., pulse trains being able to permeate the cell membrane and to facilitate the diffusion of non-permeable chemicals. The HMEC-1 cells grown in the monolayers on the chambers slides were electroporated with the perpendicular positioned parallel plate electrodes in the presence of 100 µM PI. The cellular fluorescence of PI was evaluated by photographs taken within 3 min after the applied pulse trains (Figure 1). Under non-electroporated and 0 V conditions, a small number of highly fluorescent stained cells were regularly visible. These were assumed to be non-viable cells and were often loosely attached to the culture surface. However, starting from an *E* of 100 V we observed a gradual increase of PI fluorescence in the cytosol, eventually showing more clearly the perimeters of the cells (*E*≥300 V). Correspondingly, the detection of the nuclei improved similarly along the same *E*-dependent gradient and even so with a more intensive nuclear fluorescence at higher *E*. The downer right picture shows an example of the efficiency of EP for an area between the electrodes and partly outside. Note that the left part within the electric field shows cells with a much more advanced level of PI fluorescence. These results show that the present experimental setup was suitable for efficient EP/ECT.

### Monolayer permeability

Previous permeability experiments with human umbilical vein endothelial cells grown on membrane inserts showed at 30 min after the EP a significant voltage-related increase in permeability [17]. The inserts and electrodes used by Kanthou et al. [17] enabled the HUVEC monolayers to be positioned parallel to and between the ends of the electrodes, which is different than the present perpendicular positioning of the HMEC-1 monolayers between the ends of the electrodes. In addition, the long term cell survival of electroporated HMEC-1 cell suspensions in the presence of 300 nM bleomycin was 25% [19]. Therefore, we assessed the time dependence and voltage dependence of EP or ECT on the HMEC-1 monolayer permeability.

**Figure 2:**
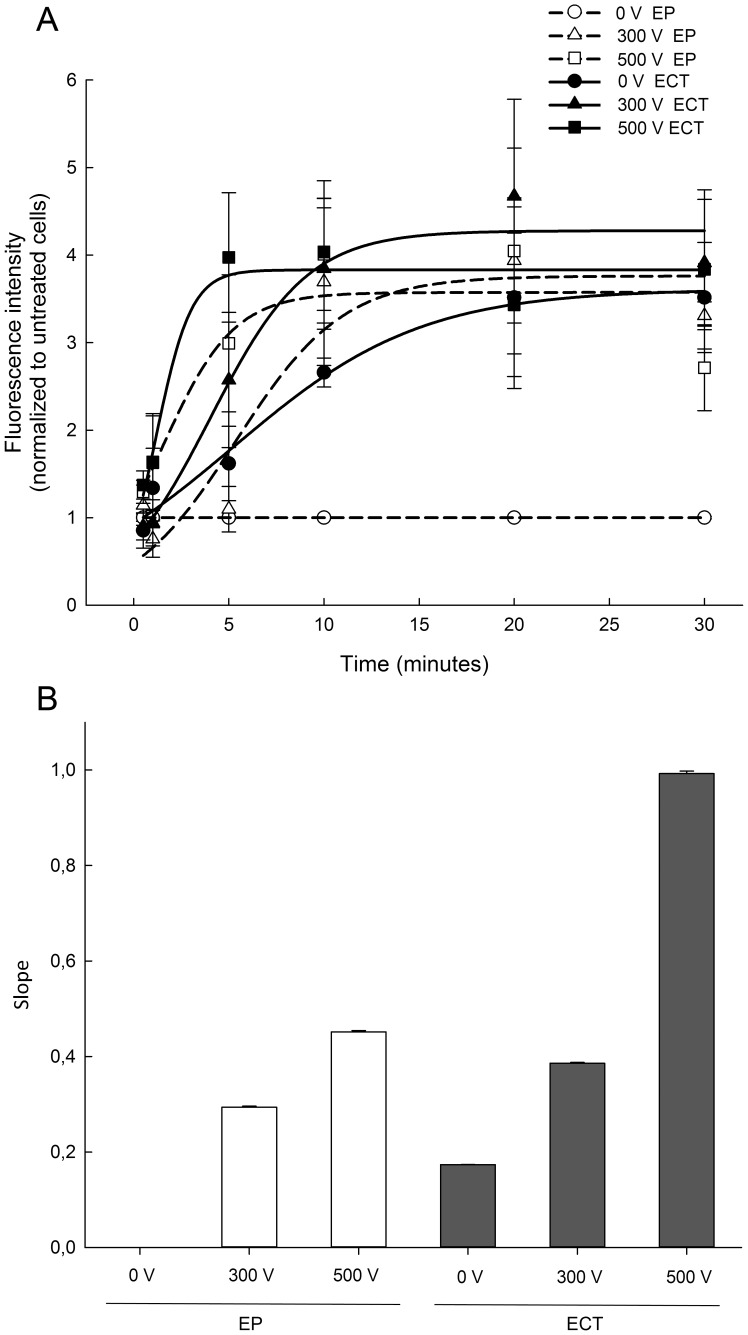
HMEC-1 monolayer permeability after exposure to electroporation or electrochemotherapy with 300 nM bleomycin. (A) Time dependent changes in fluorescence intensity normalized to the fluorescence intensity of control monolayers. Each data point represents the average fluorescence intensity ± SE from *n* = 3 independent experiments, used to fit a three-parameter sigmoidal curve. (B) The rate of permeability of the monolayer is represented by the slope of the initial linear part of the fitted three-parameter sigmoidal curve (bars represent the slope ± SE of the slope; R^2^>0.997).

Exposure of HMEC-1 monolayers to EP or ECT resulted in increased monolayer permeability. The fluorescence intensity increased in time dependent manner for each treatment and eventually reached a plateau (Figure 2A). To differentiate between different treatments more clearly, the slope of the initial linear part of each curve was used to compare the rate of permeability for each treatment (Figure 2B). ECT with 300 nM bleomycin at 300 V and 500 V significantly increased monolayer permeability rate in comparison to EP at 300 V and 500 V alone, demonstrating a more pronounced synergistic effect of bleomycin at 300 V and 500 V.

### Electrochemotherapy-induced morphology changes

**Figure 3:**
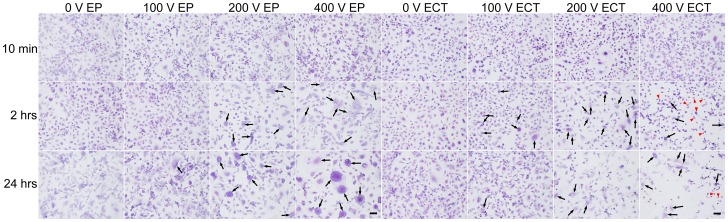
Morphology changes of electroporation- or electrochemotherapy-treated HMEC-1 monolayers. Pictures (10×) of Giemsa stained monolayers cultured on glass chamber slides at 10 min, 2 hrs and 24 hrs post-EP, respectively, with indicated *E*, either without (EP) or in the presence of 300 nM bleomycin (ECT). Bar = 100 µm, black arrow = polykaryonic/fused cells, red arrow head = spindle-like cell.

At high magnification the intact Giemsa stained HMEC-1 monolayers in the chamber slides showed endothelial cells often not densely packed, with a fair amount of intercellular space. The Giemsa stained endothelial cells displayed typical epithelial morphology with extensions (Figure 3). While 10 min after low *E* treatment no detectable cell morphology changes were observed, at *E*>200 V obvious cell swelling was observed, for both EP and ECT. At 2 hrs post-EP the perimeters of the cells were less smooth, while for the voltages ≥200 V swollen and fused cells were visible (indicated by black arrows). Besides, in the presence of bleomycin the cells suffered some decrease in turgidity, which was more obvious at >200 V where cells appeared to be shrunk, e.g. spindle-like appearance (indicated by red arrow heads). Note that at 400 V post-ECT treatment, more cells displayed spindle-like morphology. Recovery to intact monolayers appeared within 24 hrs for EP/ECT-treatments of ≤200 V. However, after EP using *E*>200 V often the monolayers showed a patchwork of cell fragments, fused into large patches of cell material, live cells, and large polykaryonic cells with the nuclei clustered centrally, demonstrating cell fusion, while for the corresponding ECT-conditions, less polykaryonic cells and besides damaged individual cells were noticed. In addition, at 2 hrs and 24 hrs post-ECT treatment at *E*<200 V fewer cells were attached to the chamber slides, indicating a pronounced cell death.

### Electrochemotherapy-induced effects on the cytoskeletal protein F-actin

**Figure 4:**
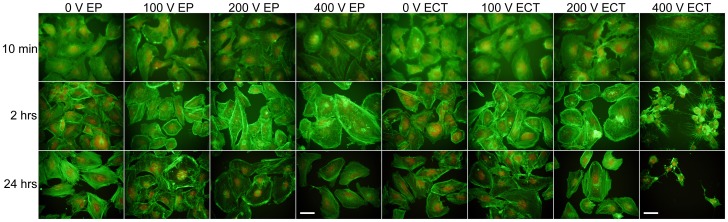
Cytoskeleton F-actin changes in electroporation- or electrochemotherapy-treated HMEC-1 monolayers. High magnification pictures (40×) of F-actin stained monolayers cultured on glass chamber slides were taken at 10 min, 2 hrs and 24 hrs post-EP with indicated *E*, either without (EP) or in the presence of 300 nM bleomycin (ECT). Green is fluorescein-conjugated phalloidin/F-actin, red is DNA/RNA stained with PI. Bar = 50 µm.

Monolayers stained with fluorescein conjugated phalloidin showed typical rigid F-actin fibers spanning the cytosol (Figure 4). Analysis of the fluorescence immediately after EP showed that these typical F-actin fibers are unaffected during *E*<200 V, but additional granules and spots appeared towards the outer membrane (brighter and denser fluorescence) during both EP and ECT. However, with higher *E* the fluorescent actin fibers turned less rigid, fragmented, and the cell membrane obtained a honeycomb-like appearance as previously observed [17]. This seemed more pronounced in the presence of bleomycin over the whole *E*-range, even at 0 V. At 2 hrs post-EP we observed cell swelling, cytosolic fusion, and recruitment of rigid F-actin fibers. These were organized into stress fibers obviously confined towards the outer membrane, and with *E*>200 V were less pronounced, even fragmented or granulated. In addition, at 2 hrs post-ECT for the higher *E* typical actin fibers disappeared, the fluorescence got more densely packed and cells appeared to be shrunk or collapsed, observed as a star-like appearance, indicating cell shrinkage. At 24 hrs post-EP/ECT after the low *E* the monolayers recovered to control levels with possible recruitment of F-actin stress fibers. For EP at higher *E* (>200 V) the cells remained enlarged (probably fused cells), while at ECT≥200 V an increase of dying cells that had degranulated and fragmented actin was observed. Thus the integrities of the monolayer were clearly affected.

### Electrochemotherapy-induced effects on the cytoskeletal protein Beta-tubulin

**Figure 5:**
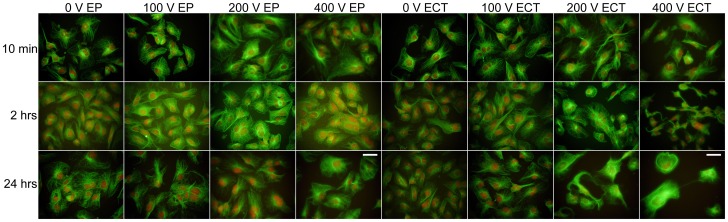
Cytoskeleton Beta-tubulin changes in electroporation- or electrochemotherapy-treated HMEC-1 monolayers. High magnification pictures (40×) of Beta-tubulin stained monolayers cultured on glass chamber slides were taken at 10 min, 2 hrs and 24 hrs post-EP with indicated *E*, either without (EP) or in the presence of 300 nM bleomycin (ECT). Green is fluorescein stained Beta-tubulin using a double immunostaining protocol, red is DNA/RNA stained with PI. Bar = 50 µm.

During control conditions the HMEC-1 monolayers stained for Beta-tubulin according to double immunocytochemistry using a secondary fluorescein-conjugated antibody showed a typical open finger lace-like thread arrangement of microtubules covering the cell body: dense in the centre, with the fiber-ends open towards the cell membrane (Figure 5). Typically, immediately post treatment and especially during higher *E* (>200 V) these lace-like structures rearranged, became more densely packed, appeared as a closing of a hand (fist-like), and subsequently fragmented. In the presence of bleomycin more closed-finger structures were present compared to EP. At 2 hrs post-EP/ECT densely packed fluorescent microtubules were observed. Also swollen cells and, concluding from the PI counterstain, merged polykaryonic cells were identified. In addition, at 2 hrs post-ECT at *E*>200 V even more densely packed microtubules were observed and thus more intense fluorescence, with the cells becoming spindle-like, and obviously collapsing and shrinking. This was further observed at 24 hrs at *E*≥200 V with cells dying. In contrast, at 24 hrs post-EP the fluorescence seemed recovered to structures as observed in control cells, with fused cells still visible at the higher *E*.

### Quantified electrochemotherapy-induced monolayer integrity changes: Covered surface area

Additional monolayers were exposed to ECT to cover the range of 3 nM to 1000 nM bleomycin concentrations, were stained with Giemsa and analysed at higher magnification to quantify changes in the covered surface area and the number of cells.

**Figure 6:**
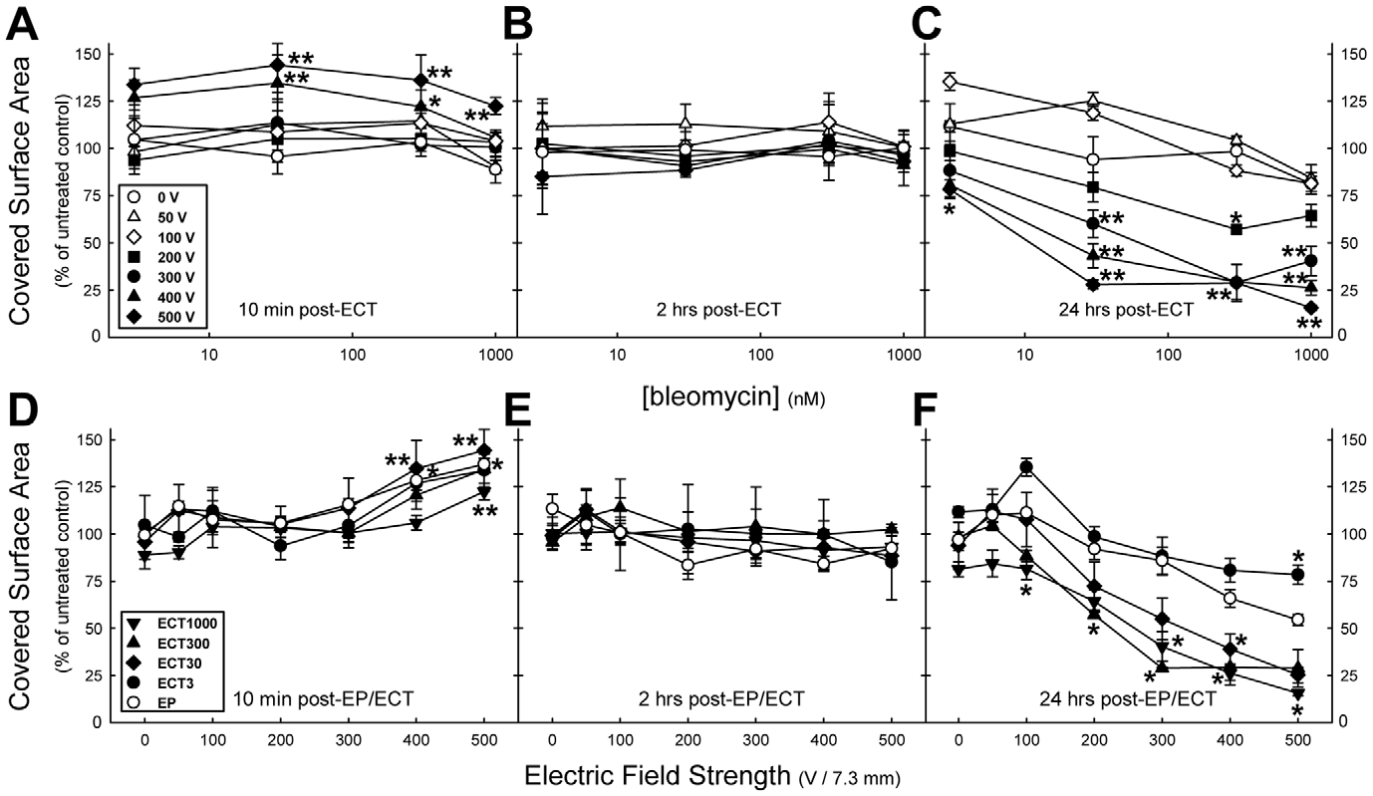
The covered surface area of electroporation- or electrochemotherapy-treated HMEC-1 monolayers. The covered surface area of the monolayers at 10 min (A, D), 2 hrs (B, E), and 24 hrs (C, F) post-exposure. Each data point represents the mean ± SE from *n* = 3 independent experiments, except for post-ECT1000 10 min *n* = 5, 24 hrs *n* = 6. *P<0.05, **P<0.001 compared to 0 V (A, B, C, D), or to EP data (E, F).

At 10 min post-ECT the covered surface areas did not show any changes over the range of 3 nM to 1000 nM bleomycin for the electric field strengths ≤300 V (Figure 6A). However, for the 400 V and 500 V treatments an increase in the covered surface area was observed (Figure 6A). This is obviously caused by the swelling of the cells due to the EP-induced fluid uptake, and is not dependent on the presence of bleomycin, as it was observed for *E*≥300 V of both EP and the various ECT treatments (≥30 nM bleomycin, Figure 6A and D). At 2 hrs post-ECT no changes in covered surface area were observed. While at 24 hrs post-treatment for each bleomycin concentration a clear separation was observed between the lower *E* (≤100 V) and the higher *E*, resulting in a voltage-dependent decrease of the covered surface area (Figure 6C). Besides, at 24 hrs post-ECT the monolayers were more severely affected than the EP-treated monolayers (Figure 3 and Figure 6F), with a clear bleomycin-dependent decrease in the covered surface area.

### Quantified electrochemotherapy-induced monolayer integrity changes: Number of cells

**Figure 7:**
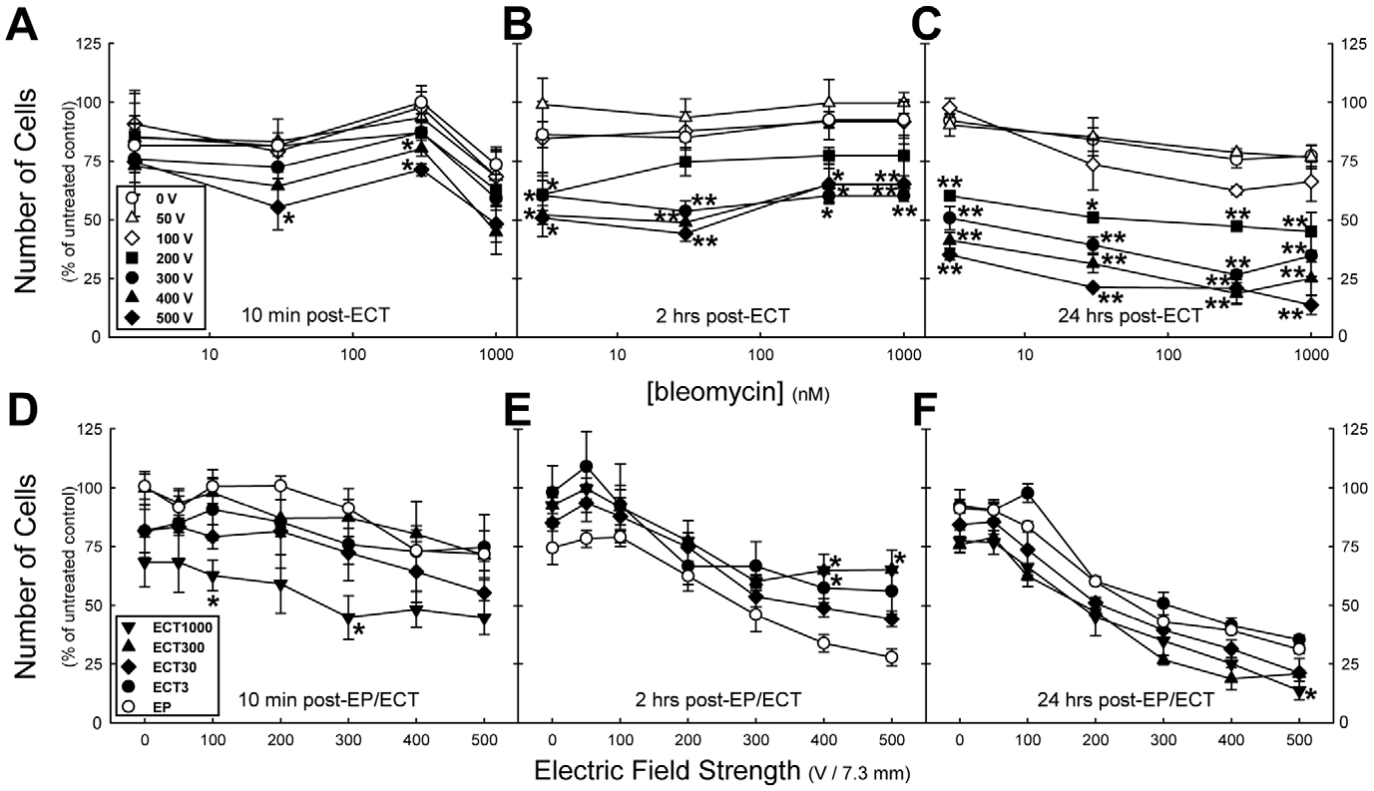
The number of cells in electroporation- or electrochemotherapy-treated HMEC-1 monolayers. The number of cells in the monolayers at 10 min (A, D), 2 hrs (B, E), and 24 hrs (C, F) post-exposure. Each data point represent the mean ± SE from *n* = 3 independent experiments, except for post-ECT1000 10 min *n* = 5, 24 hrs *n* = 6. * P<0.05, ** P<0.001 compared to 0 V (A, B, C) or to EP data (D, E, F).

The number of cells over the three time points post-exposure clearly showed different behavior for the low and high *E* (Figure 7A–C). There was no obvious effect observed among the *E*≤100 V, whereas at higher *E* a clear voltage- and concentration-dependent decrease of the number of cells in the monolayers was noticed (Figure 7B). This was even more pronounced at the 24 hrs post-ECT time point (Figure 7C). These ECT-induced effects were bleomycin-concentration dependent, and besides, contrasted with the EP effect. At 2 hrs post-treatment we observed larger number of cells during the various ECT treatments than during the EP (Figure 7E). However, as was noticed from the Giemsa staining, at this time the cells became spindle-like as if the cells shrunk. In addition, as revealed by the immunofluorescence, the cytoskeleton became first pronouncedly honeycomb-like (at 10 min), followed by star-like (at 2 hrs) as if the cytoskeleton collapsed. Consequently, at 24 hrs post-ECT the monolayers are more severely affected than the EP-treated monolayers: the number of cells showed an initial decline (*E*<100 V), combined with a clear voltage-dependent decrease that resulted in destruction of monolayers at *E*≥200 V.

## Discussion

### *In vitro* endothelial cell system to study monolayer integrity

Our results present an *in vitro* endothelial cell system in which cultured adherent endothelial cells of microvascular origin growing in monolayers were used to mimic tumour endothelium. Using perpendicular positioned electrodes we were able to effectively permeabilise the cells in the monolayer and evaluate the ECT-induced changes in monolayer permeability, cell morphology and cytoskeletal proteins. Thus, the current assay is a realistic model for elucidating mechanisms involved in ECT-induced vascular disruption in tumours and for optimizing ECT and EP treatments.

### Present results and the vascular-disrupting effect

Overall, the present data are in good agreement with the differences between EP and ECT as proposed for the time-dependent changes in local tumour blood flow and provide some further evidence about the vascular effects induced by ECT [6]. Application of EP induces a rapid, but transient two-phase decrease in blood flow. Because of vasoconstriction of afferent arterioles an almost complete shut-down of blood flow occurs instantaneously and lasts for several minutes. In the following 24 hours the microvascular endothelial barrier is transiently diminished: endothelial cells round up, decreasing the blood flow and simultaneously causing an increase of the vascular permeability. This is supposed to involve disrupted cytoskeletal structures. In addition, the presence of bleomycin (ECT) induces further damage to the microvascular endothelium, including apoptosis of the endothelial cells, and therefore leads to long-lasting and permanent decrease in local blood flow [6]. In our present study, we showed that the initially induced effect consist of cell swelling (10 min), followed at 2 hrs post-treatment by cell fusion, which for EP lasts about 24 hrs and for ECT with bleomycin results in cell death. Furthermore, we demonstrated by the immunofluorescence that both F-actin and Beta-tubulin proteins were affected. Overall, the present morphology data, and the immunofluorescence data revealed that the changes induced by ECT have an earlier onset than the changes after EP. In line with these results we demonstrated, using the monolayer permeability assessment, that ECT compared to EP clearly has an earlier onset in affecting the permeability, and besides a more pronounced effect, which is probably due to the additive effect of EP and bleomycin, as the presence of bleomycin alone (without simultaneous electric pulses) induced changes in vascular permeability, which might involve endocytosis [20]. Our results thus demonstrate earlier onset of changes induced by ECT, which were not previously demonstrated *in vivo*, most probably due to the robustness of the used methods (Patent blue staining, laser Doppler flowmetry, [5], [21]). Moreover, the present EP permeability results showed a similar *E*-dependence as the 30 min post-EP data obtained from the parallel electrodes inserts by Kanthou et al. [17]. Further, the quantified morphological changes of monolayer integrity, i.e. the covered surface area and the number of cells, at low EP/ECT electric field strengths were not significantly affected within the 24 hrs post treatment. However, for EP with *E*>150 V, the monolayer integrities did not recover up to the control level, while the threshold was generally near 100 V for the ECT treatments. Thus, at 24 hrs post-ECT the monolayers were more severely affected than the EP-treated monolayers. These results are in line with the observation that significant loss of HUVEC cell viability was detected within the hour, while within 24 hrs large *E* drastically reduced the cell viability [17].

### *In vivo* vascular-disrupting effect

It is also worth mentioning, that treatment of subcutaneous SA-1 tumours in mice with similar EP protocols as used over here showed increased blood flow at the lower voltages, while decreased blood flow and increased growth delay after the higher voltages [22]. This showed the antitumour effectiveness of EP only and the results of our study at the cellular level support the proposed model and provide further evidence of the processes involved in the vascular disrupting effect.

### Electroporation-induced cytoskeletal effects

We show here for the endothelial HMEC-1 cells that both F-actin and Beta-tubulin proteins changed during treatment. The same effects, immediate and profound disruption of both microfilament and microtubule cytoskeletal networks were demonstrated in HUVEC monolayers exposed to electric pulses [17]. These effects were *E*-dependent and reversible within the hour, although within 24 hours, after exposure to large *E* only few cells preserved the ability for complete cytoskeletal renewal [17]. In contrast to the results obtained in both endothelial cells lines, it was previously shown that the actin microfilaments remained unchanged for both electroporated suspended chicken fibroblasts [18] and monolayers of Chinese hamster ovary cells [11]. However, the microtubules were disrupted within 10 minutes post-EP, which was reversed to control within the hour [11], [18]. It is known though that the cytoskeleton organization of Chinese hamster ovary cells is very dependent on the strain [11], [23]. Recently, differential effect of EP on actin cytoskeleton was observed in breast cancer cells and fibroblasts, being more pronounced in cancer cells and only transitory in 3T3 fibroblasts [24]. Besides, it is well documented that the type of cell, the cell morphology, the cell density, the orientation within the electric field, and the pulse protocol are factors that highly influence the permeabilisation of cells and thus the changes that can be observed [25]–[33]. Although the pulse protocols of the before mentioned cytoskeleton studies are similar to ours, up till now the human microvascular endothelial cells are the closest resembling tumour vascular wall. Therefore, these results are further supporting the vascular disruption action of ECT. Namely, besides pronounced sensitivity of their cytoskeleton, the endothelial cells are the first cells exposed to bleomycin after its intravenous injection and are also exposed to higher electric field compared to the neighboring tumour and normal stromal cells [5]. Furthermore, the lack of the effect of ECT to normal tissue surrounding tumour, can be to some extent ascribed to the resistance of actin filaments to EP in fibroblasts.

### HMEC-1 cells in monolayer versus suspension

Previously, suspended HMEC-1 cells were used for both EP and ECT [19]. Although the cell survival was assessed with a MTT assay 3 days after treatment [19], as compared to 24 hrs in the present study, the *E* and the cell survival numbers of that study were both higher as compared with the present results. During EP at the highest *E* of 1800 V/cm the cell survival was measured to be about 60% [19], while presently for the HMEC-1 monolayers cell survival at the highest *E* of 685 V/cm (500 V) was 31%. For suspended ECT treated cells in the presence of 3 nM or 1 µM bleomycin at an *E* of 1800 V/cm the cell survival was measured to be 50% and 20% respectively [19]. Herein using the HMEC-1 monolayers we showed that after the highest *E* of 685 V/cm in the presence of 3 nM or 1 µM bleomycin the cell survival was 35% and 17% respectively. This demonstrates that the post-EP/ECT cell survival for the monolayers, which more closely resemble *in vivo* vascular wall, was reduced compared to the cell suspensions, concluding that the cells in endothelium are very susceptible to both EP and ECT, also due to their specific assembly consisting of interendothelial junctions.

## Conclusion

The present *in vitro* results show clear differential effects describing different changes in morphology, cytoskeleton proteins, monolayer permeability and monolayer integrity between ECT and EP for human microvascular endothelial cells. For both ECT and EP, the initial cellular damage was observed at 10 min when swollen cells and an increase in monolayer covered surface area were noticed. Besides, the formation of honeycomb-like actin bundles was observed, while the number of cells remained unchanged. The EP-induced decrease in cells and covered surface area, observed from *E*>150 V, were *E*-dependent and within 24 hrs partly recoverable. The ECT-induced cellular effects developed at 2 hrs in spindle-like cells and the observation of more densely packed F-actin and Beta-microtubulin, which were dependent on the amount of bleomycin and the *E* (>50 V). Besides, for *E*>150 V ECT the decrease in cells and covered surface area was not recoverable within 24 hrs. The effects of monolayer integrity were reflected in the enhanced monolayer permeability, with the ECT being more severe with an earlier onset. In addition, we conclude that in endothelial cell changes induced by ECT with bleomycin as compared to EP lead to quicker and more pronounced monolayer integrity damage, which provide further insight into the cellular changes of the vascular disrupting action of electrochemotherapy.
